# RCSB Protein Data Bank: Delivering integrative structures alongside experimental structures and computed structure models

**DOI:** 10.1093/nar/gkaf1187

**Published:** 2025-11-08

**Authors:** Brinda Vallat, Yana Rose, Dennis W Piehl, Jose M Duarte, Sebastian Bittrich, Chunxiao Bi, Joan Segura, Arthur Zalevsky, Monica R Sekharan, Benjamin M Webb, Charmi Bhikadiya, Maria Voigt, Jeremy Henry, Henry Chao, Aditya Pingale, David Raymond, Jared Sagendorf, Ezra Peisach, Zukang Feng, Vladimir Guranovic, James Smith, Alexander S Rose, Jasmine Y Young, Helen M Berman, Andrej Sali, Stephen K Burley

**Affiliations:** Research Collaboratory for Structural Bioinformatics Protein Data Bank and the Institute for Quantitative Biomedicine, Rutgers, The State University of New Jersey, Piscataway, NJ 08854, United States; Rutgers Cancer Institute, Rutgers, The State University of New Jersey, New Brunswick, NJ 08901, United States; Research Collaboratory for Structural Bioinformatics Protein Data Bank, San Diego Supercomputer Center, University of California, La Jolla, CA 92093, United States; Research Collaboratory for Structural Bioinformatics Protein Data Bank and the Institute for Quantitative Biomedicine, Rutgers, The State University of New Jersey, Piscataway, NJ 08854, United States; Research Collaboratory for Structural Bioinformatics Protein Data Bank, San Diego Supercomputer Center, University of California, La Jolla, CA 92093, United States; Research Collaboratory for Structural Bioinformatics Protein Data Bank, San Diego Supercomputer Center, University of California, La Jolla, CA 92093, United States; Research Collaboratory for Structural Bioinformatics Protein Data Bank, San Diego Supercomputer Center, University of California, La Jolla, CA 92093, United States; Research Collaboratory for Structural Bioinformatics Protein Data Bank, San Diego Supercomputer Center, University of California, La Jolla, CA 92093, United States; Research Collaboratory for Structural Bioinformatics Protein Data Bank, Department of Bioengineering and Therapeutic Sciences, the Quantitative Biosciences Institute (QBI), and the Department of Pharmaceutical Chemistry, University of California San Francisco, San Francisco, CA 94157, United States; Research Collaboratory for Structural Bioinformatics Protein Data Bank and the Institute for Quantitative Biomedicine, Rutgers, The State University of New Jersey, Piscataway, NJ 08854, United States; Research Collaboratory for Structural Bioinformatics Protein Data Bank, Department of Bioengineering and Therapeutic Sciences, the Quantitative Biosciences Institute (QBI), and the Department of Pharmaceutical Chemistry, University of California San Francisco, San Francisco, CA 94157, United States; Research Collaboratory for Structural Bioinformatics Protein Data Bank, San Diego Supercomputer Center, University of California, La Jolla, CA 92093, United States; Research Collaboratory for Structural Bioinformatics Protein Data Bank and the Institute for Quantitative Biomedicine, Rutgers, The State University of New Jersey, Piscataway, NJ 08854, United States; Research Collaboratory for Structural Bioinformatics Protein Data Bank, San Diego Supercomputer Center, University of California, La Jolla, CA 92093, United States; Research Collaboratory for Structural Bioinformatics Protein Data Bank and the Institute for Quantitative Biomedicine, Rutgers, The State University of New Jersey, Piscataway, NJ 08854, United States; Research Collaboratory for Structural Bioinformatics Protein Data Bank and the Institute for Quantitative Biomedicine, Rutgers, The State University of New Jersey, Piscataway, NJ 08854, United States; Research Collaboratory for Structural Bioinformatics Protein Data Bank and the Institute for Quantitative Biomedicine, Rutgers, The State University of New Jersey, Piscataway, NJ 08854, United States; Research Collaboratory for Structural Bioinformatics Protein Data Bank, Department of Bioengineering and Therapeutic Sciences, the Quantitative Biosciences Institute (QBI), and the Department of Pharmaceutical Chemistry, University of California San Francisco, San Francisco, CA 94157, United States; Research Collaboratory for Structural Bioinformatics Protein Data Bank and the Institute for Quantitative Biomedicine, Rutgers, The State University of New Jersey, Piscataway, NJ 08854, United States; Research Collaboratory for Structural Bioinformatics Protein Data Bank and the Institute for Quantitative Biomedicine, Rutgers, The State University of New Jersey, Piscataway, NJ 08854, United States; Research Collaboratory for Structural Bioinformatics Protein Data Bank and the Institute for Quantitative Biomedicine, Rutgers, The State University of New Jersey, Piscataway, NJ 08854, United States; Research Collaboratory for Structural Bioinformatics Protein Data Bank and the Institute for Quantitative Biomedicine, Rutgers, The State University of New Jersey, Piscataway, NJ 08854, United States; Mol* Consortium, San Diego, CA, United States; Research Collaboratory for Structural Bioinformatics Protein Data Bank and the Institute for Quantitative Biomedicine, Rutgers, The State University of New Jersey, Piscataway, NJ 08854, United States; Research Collaboratory for Structural Bioinformatics Protein Data Bank and the Institute for Quantitative Biomedicine, Rutgers, The State University of New Jersey, Piscataway, NJ 08854, United States; Department of Chemistry and Chemical Biology, Rutgers, The State University of New Jersey, Piscataway, NJ 08854, United States; Department of Quantitative and Computational Biology, University of Southern California, Los Angeles CA 90089, United States; Research Collaboratory for Structural Bioinformatics Protein Data Bank, Department of Bioengineering and Therapeutic Sciences, the Quantitative Biosciences Institute (QBI), and the Department of Pharmaceutical Chemistry, University of California San Francisco, San Francisco, CA 94157, United States; Research Collaboratory for Structural Bioinformatics Protein Data Bank and the Institute for Quantitative Biomedicine, Rutgers, The State University of New Jersey, Piscataway, NJ 08854, United States; Rutgers Cancer Institute, Rutgers, The State University of New Jersey, New Brunswick, NJ 08901, United States; Research Collaboratory for Structural Bioinformatics Protein Data Bank, San Diego Supercomputer Center, University of California, La Jolla, CA 92093, United States; Department of Chemistry and Chemical Biology, Rutgers, The State University of New Jersey, Piscataway, NJ 08854, United States; Rutgers Artificial Intelligence and Data Science (RAD) Collaboratory, Rutgers, The State University of New Jersey, Piscataway, NJ 08854, United States

## Abstract

The Protein Data Bank (PDB) archives 3D structures of macromolecules determined experimentally using various methods. It is jointly managed by the Worldwide Protein Data Bank (wwPDB) consortium. Research Collaboratory for Structural Bioinformatics (RCSB) PDB, the US data center for the PDB, provides streamlined access to >240 000 structures through a variety of research-focused tools on RCSB.org. In addition, RCSB.org makes available over 1 million computed structure models (CSMs) predicted using deep learning methods and archived in the AlphaFold Database and ModelArchive. The PDB-IHM system was developed as a wwPDB project based on community recommendations to archive structures determined using integrative/hybrid methods (IHM). These structures are computed by combining information from multiple experimental and computational techniques to overcome the limitations of traditional single methods (e.g. macromolecular crystallography, 3D electron microscopy, nuclear magnetic resonance spectroscopy). In 2024, PDB-IHM was unified with the PDB to archive integrative structures alongside single-method experimental structures. These integrative structures have been made accessible via the RCSB.org website, facilitating efficient delivery of IHM data to a broad community of PDB users. Herein, we describe the expanded capabilities of RCSB.org that support discovery, analysis, and visualization of integrative structures together with single-method experimental structures and CSMs.

## Graphical Abstract

**Graphical Abstract:**
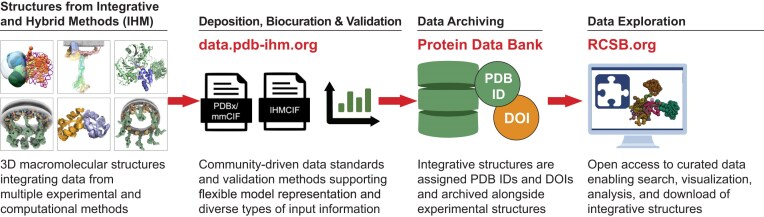

## Introduction

The Protein Data Bank (PDB) is the single global repository for experimentally determined 3D atomic structures of biomolecules and their complexes [1, 2]. Managed by the worldwide Protein Data Bank (wwPDB) organization [3], PDB promotes the FAIR (Findable, Accessible, Interoperable, and Reusable) [4] and FACT (Fairness, Accuracy, Confidentiality, and Transparency) [5] principles of scientific data management. The PDB primarily archives structures determined by macromolecular crystallography (MX), nuclear magnetic resonance (NMR) spectroscopy, and 3D electron microscopy (3DEM). Since its establishment in 1971, the archive has grown >30 000-fold and currently holds >240 000 3D biostructures that are expertly curated and rigorously validated based on community guidelines.

The Research Collaboratory for Structural Bioinformatics (RCSB) PDB [6] is the US data center for the wwPDB. It provides access to PDB data through the research-focused RCSB.org web portal. RCSB.org offers a variety of tools and features that support searching, browsing, visualizing, validating, and analyzing macromolecular structures. As a living digital resource, RCSB.org is updated weekly to incorporate new structures and enrich our understanding of every structure in the archive with annotations from ∼50 trusted external resources. In 2022, we expanded RCSB.org to provide access to ∼1 million computed structure models (CSMs) from AlphaFold Protein Structure Database (AlphaFoldDB) [7] and the ModelArchive [8]—including complete proteomes of model organisms and select pathogens—thereby enhancing the availability of 3D biostructure data to the scientific community [9].

Advances in structure determination led to the development of integrative/hybrid methods (IHM) that combine information from multiple experimental and computational sources [10]. These sources can include 3DEM, small-angle scattering (SAS), chemical crosslinking mass spectrometry (crosslinking-MS), Förster resonance energy transfer (FRET), electron paramagnetic resonance (EPR) spectroscopy, and many others. In addition, available structures of assembly components can be used as starting models. Integrative methods are complementary to traditional structure determination methods and are typically used to determine structures of larger macromolecules or complex assemblies that may be difficult or impossible to resolve with a single technique. Examples of macromolecular complexes studied using integrative approaches include the structure of the human LINE-1 ORF2p protein [11], the structure of the human SNAPc-DNA [12], and the assembly pathway of the human nuclear pore complex [13, 14].

Based on community recommendations made by the wwPDB IHM Task Force [15, 16], the PDB-Dev system was developed for deposition, biocuration, validation, and dissemination of integrative structures [17, 18]. This prototype system was built alongside the wwPDB OneDep system [19] that handles experimental structures archived in the PDB. In 2024, PDB-Dev was unified with the PDB and rebranded as PDB-IHM to denote the “IHM” branch of the PDB archive [20]. Importantly, integrative structures are now issued PDB accession codes and archived alongside experimental structures in the PDB. Following unification, RCSB.org capabilities were extended to support dissemination of integrative structures. Herein, we describe the new functionalities of RCSB.org that provide expanded access to integrative structures together with single-method experimental structures and CSMs, thus making it a comprehensive resource for 3D macromolecular structure data.

## Results and discussion

With the increasing application of integrative methods, the structural biology community has encouraged the PDB to create robust mechanisms for archiving, validating, and disseminating integrative structures in a FAIR manner [15, 16]. Archiving integrative structures introduced additional requirements when compared to single-method experimental structures in the PDB archive to accommodate new data representations and metadata:

1. Integrative structures can be multiscale, including both atomic and coarse-grained representations.
2. Integrative structures can be multistate, corresponding to multiple conformational and compositional states jointly satisfying the input restraints. In addition, the multiple states can be ordered to depict a pathway, such as an enzymatic reaction or a biochemical pathway.
3. Integrative modeling can result in a collection of models (ensembles), where each model independently satisfies the input restraints within an acceptable threshold.
4. Input restraints used in integrative modeling can be of diverse types, ranging from pairwise distances between atoms, residues, or domains fit to 2D images and 3D volumes.
5. Starting structure models of system components can come from experimental, computational, or integrative methods, and can be rigid or flexible during modeling.
6. Integrative approaches make use of many different modeling algorithms and software tools.

Accordingly, archiving integrative structures required creation of new data standards, tools, and workflows that satisfy the above requirements, capture the provenance information for all input data, enable automated data processing, support rigorous assessment of these structures, and facilitate efficient data dissemination to a broad community of users. In the following sections, we describe aspects of PDB-IHM infrastructure and extensions of the RCSB.org web portal that accomplish these goals.

### PDB-IHM data deposition, biocuration, and validation

The foundational data standard for archiving integrative structures is provided by IHMCIF [21], an extension of the PDBx/mmCIF data dictionary [22, 23] used by the PDB. At its core, IHMCIF follows the same set of definitions for representing 3D atomic structures of macromolecules as PDBx/mmCIF. Information specific to integrative structure determination, such as definitions for multiscale, multistate, and ordered-state structural models, spatial restraints from various experimental data types (e.g. 3DEM, crosslinking-MS, FRET, SAS, EPR), starting structure models used, and references to experimental data archived in external repositories, are defined in the IHMCIF extension.

**Figure 1.**
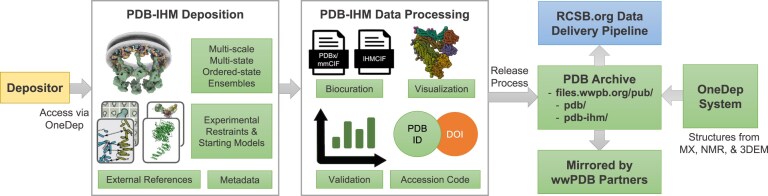
The PDB-IHM data pipeline supports (i) deposition of integrative structures, associated experimental restraints and starting models, references to related data in external resources, and other metadata details; (ii) data processing steps for manual and automated biocuration, structure validation, visualization, and issue of PDB accession codes and digital object identifiers (DOIs); and (iii) release process to the PDB archive. The PDB-IHM deposition interface can be accessed via the wwPDB OneDep system and works in parallel to OneDep, which handles structures from MX, NMR, and 3DEM. Bulk deposition via an Application Programming Interface (API) is also supported. Integrative structures archived in the PDB along with experimental structures are mirrored by the wwPDB partners and are the primary source of data for the RCSB.org data delivery pipeline.

The PDB-IHM system for deposition, biocuration, and validation (data.pdb-ihm.org; Fig. 1) relies on the PDBx/mmCIF and IHMCIF data standards and related tools for processing integrative structures. It can be accessed through and operates in parallel with the wwPDB OneDep system and was built using the DERIVA scientific asset management software [24, 25]. The system supports harvesting and deposition of heterogeneous data and metadata associated with integrative structure modeling investigations [18, 20].

PDB-IHM biocuration includes both automated and manual steps to ensure data consistency, completeness, and compliance with archive requirements, similar to OneDep biocuration [26]. Once the process is completed, PDB accession codes are issued, and validation reports are generated. Methods for validating integrative structures have been developed [27] based on recommendations from the wwPDB IHM Task Force [15, 16] and in collaboration with community experts. A validation software package has been implemented within the IHM deposition and curation pipeline, so that users can obtain full and summary versions of validation reports along with accession codes and use them as part of the manuscript review process.

### Unification of PDB-IHM with the PDB archive

Although PDB-IHM (pdb-ihm.org) [20] was developed in parallel with OneDep, the eventual goal was to unify it with the PDB such that integrative structures are archived alongside single-method experimental structures. In 2024, unification was completed, and integrative structures are now provided under the “pdb_ihm” directory within the PDB archive [28].

Following unification, integrative structures can be downloaded from the PDB archive, which is available across multiple wwPDB partner sites, including RCSB.org, pdbe.org, and pdbj.org. Repository contents include mmCIF files, validation reports, and files reporting archive holdings. Within mmCIF files, a specific data item called “struct.structure_determination_methodology” is populated as “integrative” to clearly identify structures determined using integrative methods. Both new and pre-existing integrative structures are now identified with PDB accession codes (previously issued PDB-Dev accession codes are preserved in the “database_2” mmCIF category for provenance).

The PDB-IHM data release workflow further ensures timely delivery of updated/released IHM entries via the PDB archive, synchronously with the weekly update process for single-method PDB structures. New integrative structures are issued DOIs based on the PDB accession codes and the corresponding DOI landing pages are made available on the wwPDB.org website. DOIs allow online versions of primary publications to be cross-referenced to every structure stored in the PDB archive.

### Incorporation of integrative structures into the RCSB.org data delivery pipeline

**Figure 2.**
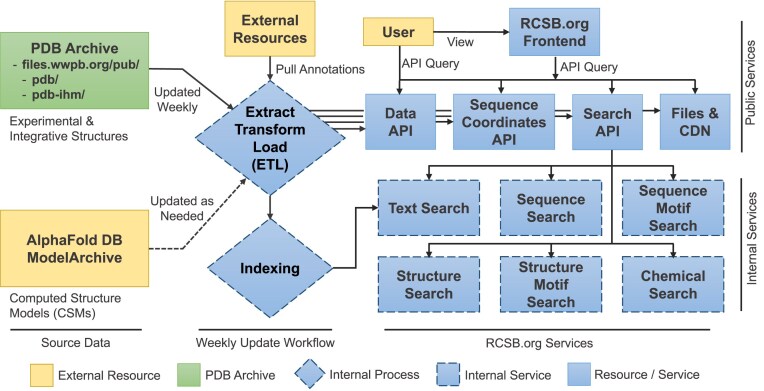
Overview of the RCSB.org modular architecture and data flow. Experimental and integrative structures are obtained from the PDB archive weekly, while CSMs are sourced from AlphaFoldDB and ModelArchive on an as-needed basis and stored on our servers. Annotations obtained from ∼50 external resources are integrated with the source data through an Extract–Transform–Load process 2, which automates data retrieval, standardization, and incorporation of heterogeneous datasets into the RCSB.org data warehouse. The front-end web portal integrates with multiple back-end services, including content delivery via a Content Delivery Network (CDN) that distributes static assets such as images and files; metadata access through the Data API (GraphQL and Representational State Transfer (REST) endpoints at data.rcsb.org); search operations through the Search API (REST endpoints at search.rcsb.org); and sequence annotation delivery through the Sequence Coordinates API (GraphQL endpoints at sequence-coordinates.rcsb.org). These services are supported by a suite of dedicated APIs and microservices.

Following the unification of PDB-IHM with PDB and the availability of integrative structures in the PDB archive, RCSB.org was extended to support parallel delivery of integrative structures. In 2022, a similar extension was carried out to incorporate ∼1 million CSMs [29] from AlphaFoldDB [7] and the ModelArchive [8]. We followed a similar approach to incorporate integrative structures into the RCSB.org data delivery pipeline (Fig. 2).

Data management is based on a schema-first approach, wherein every step of the data delivery pipeline together with supporting services and tools is informed by metadata encoded in a standard JSON (JavaScript Object Notation) schema representation [30]. These schemas are derived from the PDBx/mmCIF definitions for single-method PDB structures and the ModelCIF [31] definitions for CSMs. We extended the RCSB.org schemas to include the IHMCIF extension dictionary [21], which allows information to be extracted and consumed from IHMCIF data files. Additional definitions were introduced to the internal RCSB.org schema to support efficient search and data delivery. For example, the “rcsb_entry_info” category was extended with attributes that identify features specific for integrative structures, such as multiscale, multistate, ordered-state, and/or ensemble representations.

The RCSB.org weekly update pipeline has been extended to support PDB-IHM data, including modifications of back-end tools for extracting, transforming, and loading data into the RCSB.org data warehouse, plus libraries for integrating annotations from ∼50 trusted external biodata resources. PDB accession codes serve as unique identifiers for integrative structures. Primary and computed data, together with external annotations, are processed and loaded for a representative model in a manner consistent with single-method experimental structures. If an entry contains multiple structure models (e.g. ensemble), then Depositor(s) can select one of these models to be the representative conformation. If provided by the Depositor(s), the representative model is identified based on annotations in the mmCIF file; otherwise, it is chosen as the first model containing the largest number of molecules. Moreover, provenance information is explicitly captured (via the “rcsb_entry_info.structure_determination_methodology” data item) to distinguish integrative structures from single-method experimental structures and CSMs. Finally, back-end workflows for indexing, sequence clustering, image generation, and BinaryCIF [32] file generation were extended to fully support integrative structures, including multiscale structures with coarse-grained representations.

The RCSB.org user interfaces, data delivery, and search services are supported by a set of modular microservices [30] (Fig. 2). The front-end service, accessible via the RCSB.org web portal, integrates with multiple back-end API services. Coordinate files in text and binary formats are distributed through the Files Download service (files.rcsb.org) and ModelServer API (models.rcsb.org), while static content, such as images, is delivered via a CDN. Structured metadata are available programmatically through the Data API (data.rcsb.org), which offers both REST and GraphQL endpoints. Search operations are supported by the Search API (search.rcsb.org), which offers a unified interface to heterogeneous search modalities, including 3D structure and sequence similarity searches, 3D, and sequence motif searches, and chemical structure searches—each supported by a dedicated microservice. Sequence alignments and annotations are served through the Sequence Coordinates API (sequence-coordinates.rcsb.org). All single-method experimental structures and integrative structures released in the archive are immediately accessible via the API services and RCSB.org, which are updated in synchrony with the weekly data releases every Wednesday at 00:00 Coordinated Universal Time.

### RCSB.org functionalities supporting integrative structures

**Figure 3.**
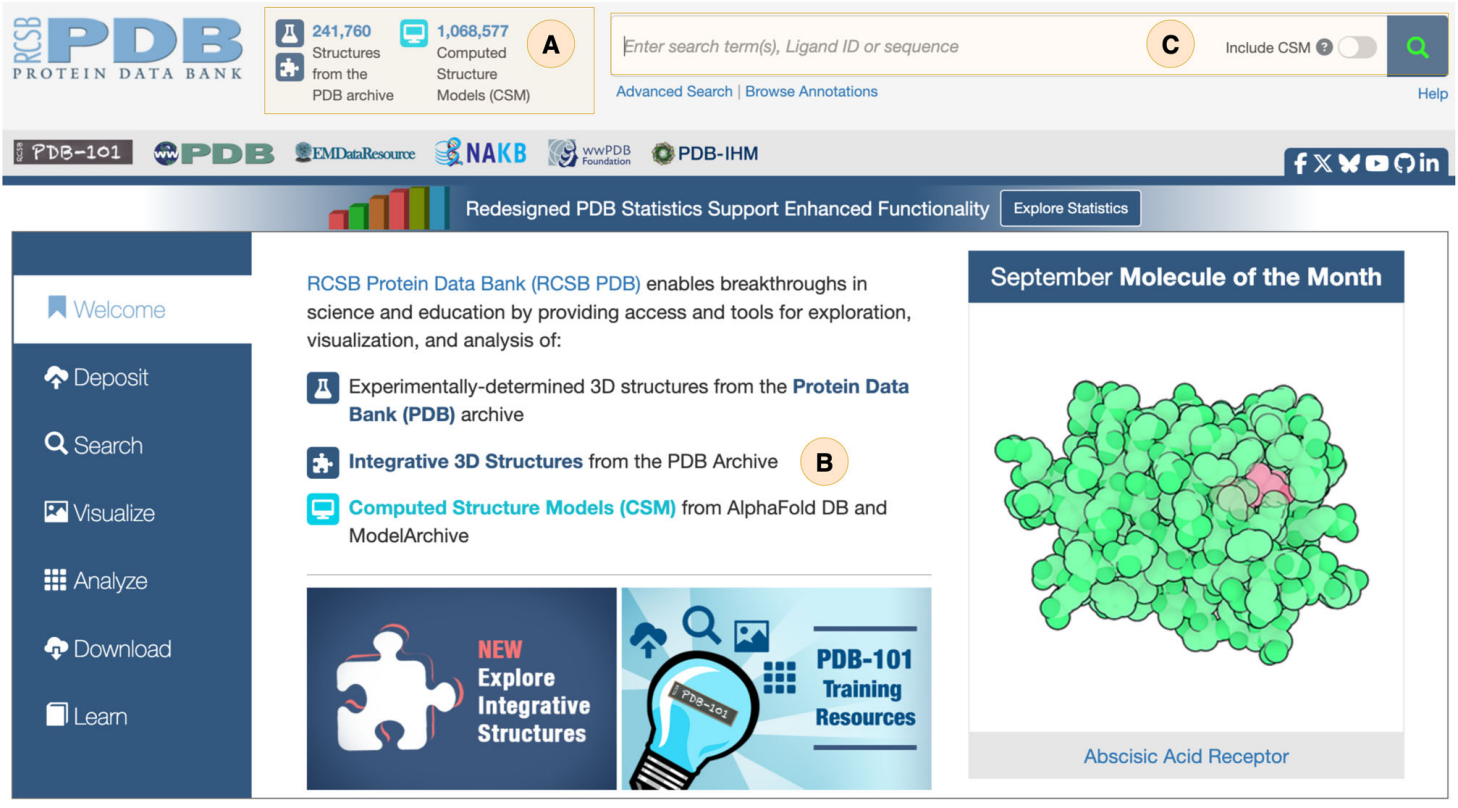
RCSB.org homepage (as of September 2025). (**A**) Display of structure counts accompanied by dedicated icons that distinguish structure types: dark-blue “puzzle piece” for integrative structures, dark-blue “Erlenmeyer flask” for experimentally determined structures, and light-blue “computer monitor” for CSMs. Clicking on these icons launches searches across the entire corresponding datasets. (**B**) Link to supporting documentation that provides guidance on data access and search functionality for integrative structures. (**C**) Keyword-based search bar for full-text queries.

The extended RCSB.org web portal provides comprehensive support for the discovery, exploration, validation, and analysis of integrative structures, fully incorporated into the website search, navigation, and visualization functionalities. From the homepage, the total count for the PDB archive reflects both single-method experimental structures and integrative structures (Fig. 3A). To ensure clear visual distinction among structure types, consistent use of icons and colors conveys data provenance across RCSB.org: a dark-blue puzzle piece icon represents the integrative structures, a dark-blue Erlenmeyer flask icon denotes the single-method experimental structures, and a light-blue computer monitor icon denotes CSMs. Search links associated with each icon type allow direct access to the corresponding dataset. In addition, the RCSB.org homepage provides a link to supporting documentation with guidance on data access and search functionality for integrative structures (Fig. 3B).

**Figure 4.**
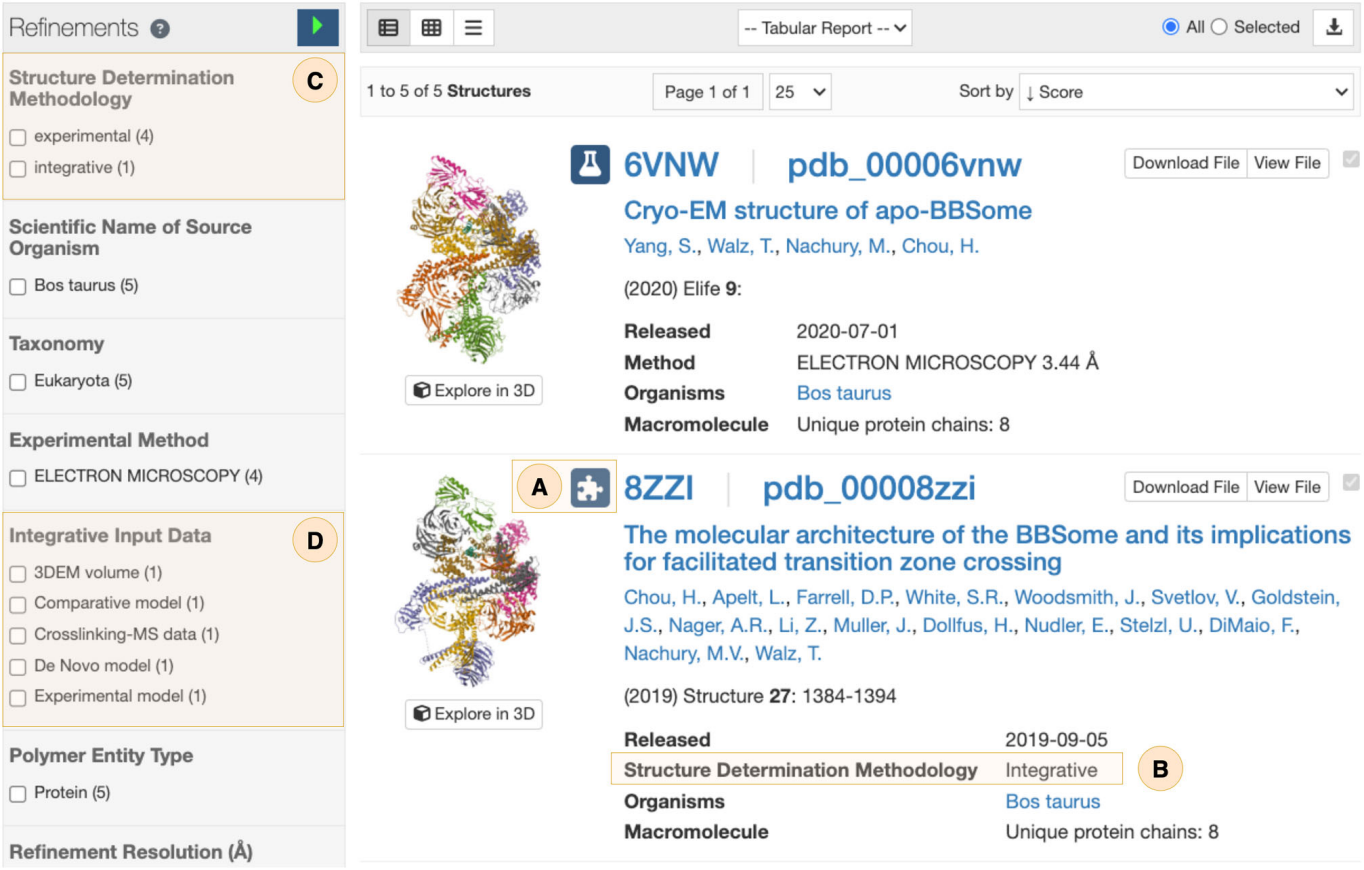
Search results page displaying both experimental and integrative structures. (**A**) Distinct structure-type icons: a puzzle piece for integrative structures and a flask for experimentally determined structures. (**B**) Search result snippets for integrative structures explicitly report the *Structure determination methodology* used. Refinement option for filtering results by (**C**) *Structure determination methodology* and (**D**) *integrative input data*.

Integrative structures can be accessed via keyword-based searches (Fig. 3C), which return both single-method experimental and integrative structures (Fig. 4). Search result snippets for integrative structures are explicitly marked with a puzzle-piece icon (Fig. 4A) and report the structure determination methodology (Fig. 4B), ensuring that users are immediately informed of the provenance. Faceted filters, including *Structure determination methodology* (Fig. 4C) and *Integrative input data* (Fig. 4D), allow refinement of search results to exclusively display integrative structures, facilitating targeted exploration of specific modeling approaches.

**Figure 5.**
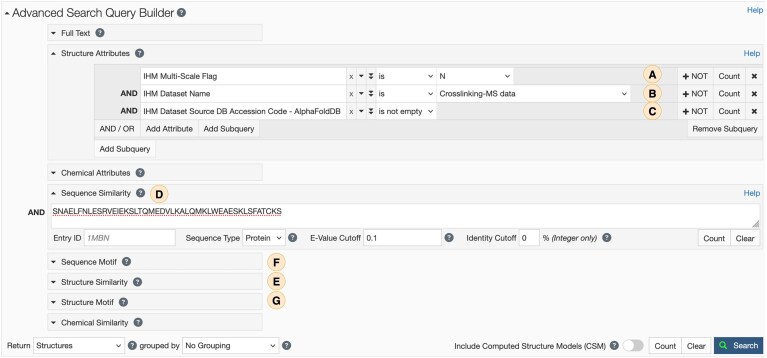
Example query in the Advanced Search interface for integrative structures. The query combines metadata-based criteria, including (**A**) atomic resolution integrative structures that (**B**) utilize crosslinking-MS experimental data and (**C**) AlphaFoldDB *de novo* models as starting structures, with (**D**) sequence-based searches. Additional scientific searches, such as (**E**) 3D structure similarity and motif-based (**F**) sequence or (**G**) structure searches, can also be applied to further refine results.

**Figure 6.**
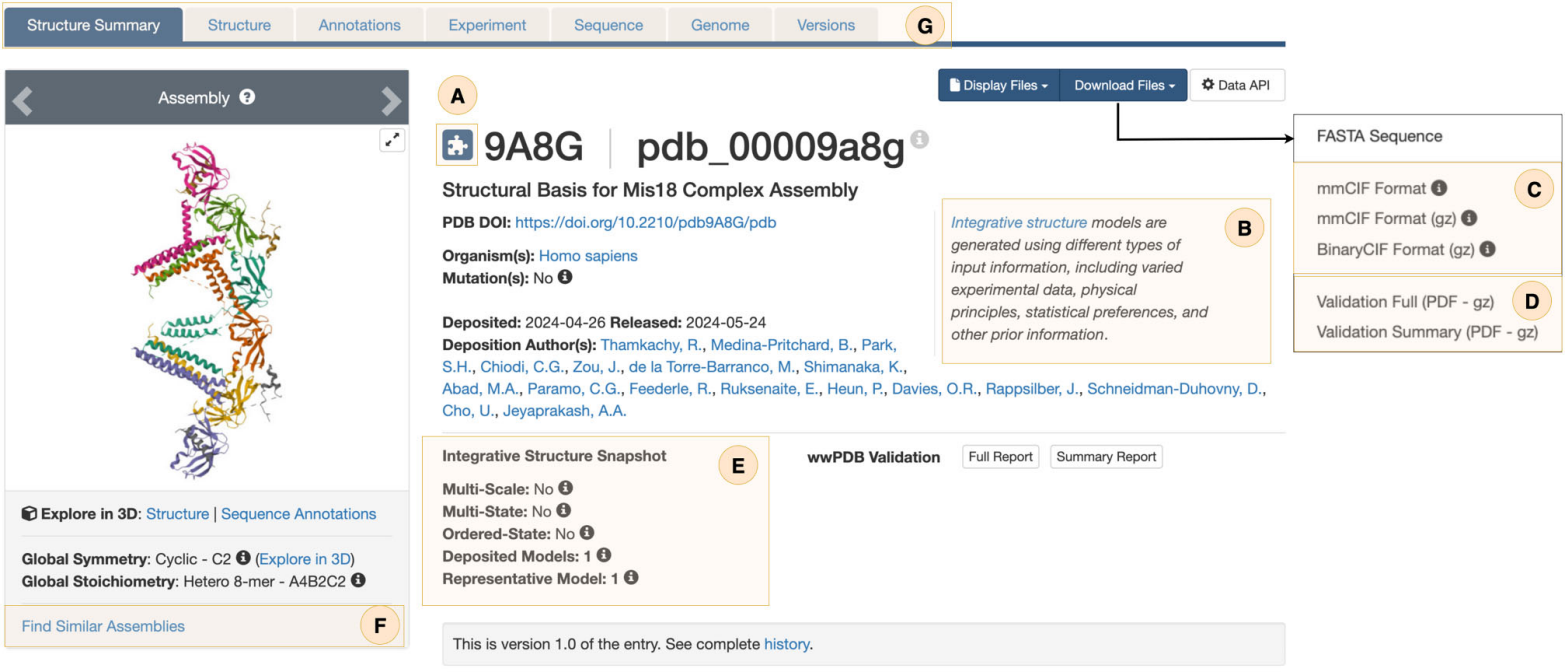
SSP for the integrative structure of the Mis18 complex (PDB ID: pdb_00009a8g) [33]. (**A**) A puzzle piece icon used to identify integrative structures. (**B**) An explanatory note describing integrative structures. (**C**) Coordinate data available for download in mmCIF and BinaryCIF formats. (**D**) Validation reports provided in PDF format (full and summary). (**E**) High-level model features (e.g. multiscale, multistate, ordered) and indication of the currently loaded model. (**F**) Link to launch a 3D similarity scientific search. (**G**) Specialized tabs provide focused views on different aspects of a structure.

The Advanced Search interface (rcsb.org/search/advanced) offers fine-grained control for working with integrative structures (Fig. 5). Search parameters include model features (e.g. multiscale, multistate, ordered-state models) (Fig. 5A), types of input experimental data (e.g. crosslinking–MS, 3DEM maps, NMR restraints) (Fig. 5B), and associated dataset accessions from external repositories, including Biological Magnetic Resonance Data Bank (BMRB) [34], Electron Microscopy Data Bank (EMDB) [35], Small-angle Scattering Biological Data Bank (SASBDB) [36], and PRoteomics IDEntifications Database (PRIDE) [37] (Fig. 5C). In addition to these metadata-based criteria, Advanced Search supports scientific queries, including sequence similarity (Fig. 5D), 3D structure similarity (Fig. 5E), and motif-based sequence or structural searches (Fig. 5F and G). These capabilities allow retrieval of integrative structures that meet precisely defined criteria.

Each integrative structure is presented through its own Structure Summary Page (SSP) that provides an overview of the representative model, associated metadata, and supporting experimental data. To facilitate recognition, each page is marked with a dedicated icon identifying integrative structures (Fig. 6A) and an explanatory note describing integrative structures (Fig. 6B). Coordinate data are available for download in both mmCIF and BinaryCIF formats compatible with the IHMCIF dictionary (Fig. 6C). Two types of validation reports are provided in the PDF format: a full report and a summary report (Fig. 6D). SSPs further illuminate high-level model features, such as whether the model is multiscale, multistate, and/or ordered-state, along with a clear indication of the currently loaded representative model (Fig. 6E). In addition, users are provided with direct links to launch similarity-based searches (Fig. 6F).

**Figure 7.**
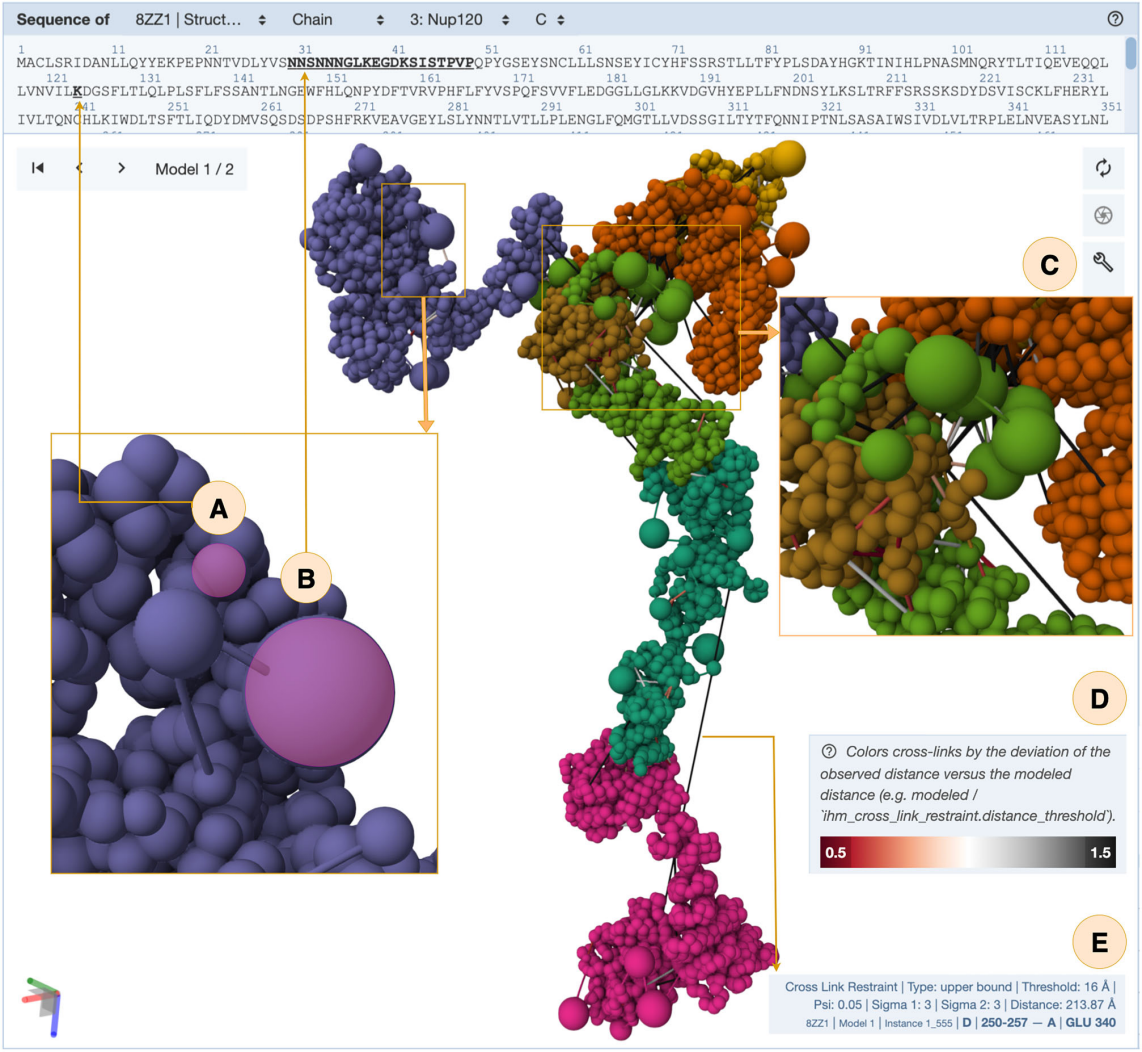
Visualization of the Nup84 sub-complex of the Nuclear Pore Complex (PDB ID: pdb_00008zz1) [38] in the Mol* viewer. Multiscale representation, with coarse-grained spheres corresponding to (**A**) single residues and (**B**) residue ranges. (**C**) Visualization of crosslinked residues connected by lines. (**D**) Crosslink restraints that are bound by an upper limit are color-coded according to the deviation between the experimentally derived input restraints used for modeling and the distances observed in the deposited model. (**E**) Hovering over a restraint visualization displays detailed information about the crosslink restraint parameters (e.g. type, threshold, uncertainties Ψ, σ₁, and σ₂, and distance).

**Figure 8.**
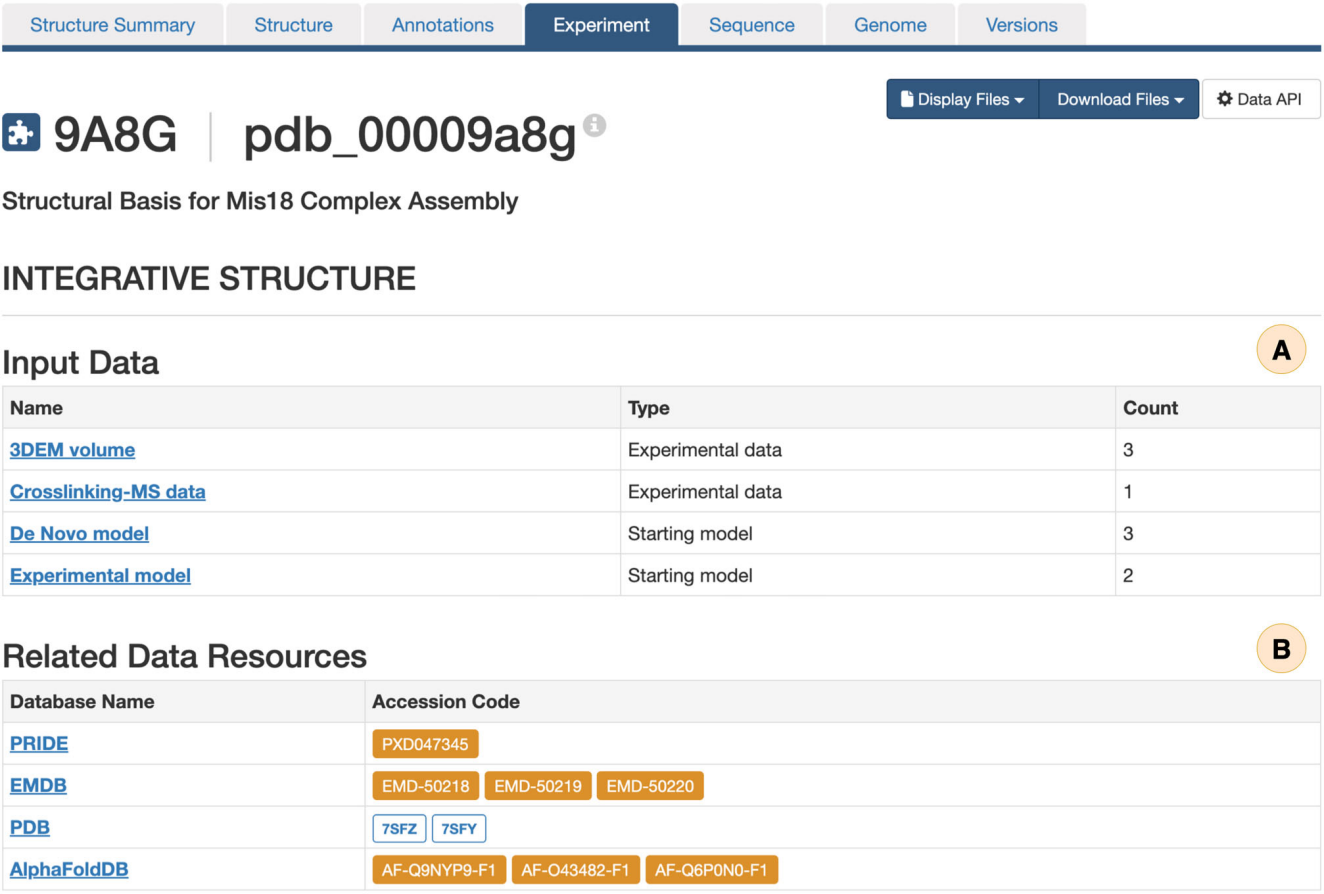
Experimental details for the integrative structure of the Mis18 complex (PDB ID: pdb_00009a8g). (**A**) Input experimental datasets used for modeling. (**B**) Related datasets deposited in external repositories.

SSPs are organized into specialized tabs (Fig. 6G): Structure tab—interactive visualization of the integrative structures via the Mol* viewer [39] directly within the web browser, with support for displaying multiscale assemblies that combine atomic and coarse-grained representations (Fig. 7A and B). In addition to these representations, crosslink restraint data can be displayed, with the option to color-code restraints according to differences between the experimental input restraints and observed distances in the deposited structure, enabling immediate visual assessment of the agreement between model and underpinning data (Fig. 7C and D). Hovering over a restraint visualization displays detailed information about the crosslink restraint parameters (e.g. type, threshold, uncertainties Ψ, σ₁, and σ₂, [40], and distance) (Fig. 7E). Annotations tab—cross-references to external biological resources. Experiment tab—a summary of input datasets used in modeling (e.g. 3DEM maps, crosslinking-MS restraints, and starting models), together with links to related data in EMDB, PRIDE, and other repositories (Fig. 8). Sequence tab—mappings of sequence features to structural elements, including domains and functional annotations, with interactive visualization bridging sequence and structure views.

Integrative structures are also included in “Groups” pages on RCSB.org [41] that cluster experimental structures, integrative structures, and CSMs based on sequence similarity or mapping to reference UniProt [42] sequences. This unified framework allows side-by-side comparison of 3D information from different sources. Moreover, the pairwise structure alignment tool [43] enables quantitative and visual comparison of atomic integrative structures, single-method experimental structures, and CSMs. Alignments can be performed for any structure or UniProt accession, supporting cross-methodology structural comparison and analysis.

## Conclusions and future perspectives

Unifying PDB-IHM with the PDB archive and making integrative structures accessible via RCSB.org has broadened the scope and capabilities of the PDB. Given the growing importance of integrative modeling in elucidating 3D structures of larger biomolecules and macromolecular complexes, these efforts address important community requirements and support sustained growth of the archive.

Upcoming developments include the creation of PDB-IHM beta archive alongside the PDB beta archive as planned by wwPDB. The beta archive will support extended PDB IDs, which will become the primary IDs when four-character accession codes are consumed (expected by 2028). We are also working on delivering IHM validation data in mmCIF format so that software tools can consume this information. Furthermore, we are working on streamlining data exchange with EMDB and PRIDE data resources to access pre-release experimental data for structure validation.

Results from a preliminary analysis carried out using artificial intelligence tools relying on large language models (LLMs) estimate that roughly 25% of lower-resolution 3DEM structures (<3.5 Å) deposited into the PDB archive as single-method experimental structures are likely to be integrative structures (we acknowledge that this metric may be an overestimate). The OpenAI GPT-3.5-turbo LLM was provided with publicly available primary publications from 2023 reporting lower-resolution 3DEM structures archived in the PDB and was tasked with identifying structures that use both a 3DEM map and other types of experimental data supported by PDB-IHM, such as crosslinking-MS. PDB structures thus identified are currently not annotated as integrative structures, and some of the data used to generate them are not archived in the PDB (i.e. there has been incomplete data capture). PDB-IHM provides a robust mechanism for deposition, biocuration, validation, and archiving integrative structures together with all the supporting data and metadata details regarding the input restraints, starting models, and modeling methods used, thus enabling FAIR data delivery. In addition, the PDB-IHM pipeline facilitates enhanced structure validation based on spatiotemporal restraints and starting models used in structure determination. Thus, we encourage the structural biology community to deposit integrative structures using the PDB-IHM.org deposition tool accessible from the wwPDB OneDep system, especially 3DEM-based integrative structures. Depositors wishing to correct the scientific record and ensure complete data capture are strongly encouraged to re-deposit their 3DEM-crosslinking-MS (or similar) integrative structures using the PDB-IHM deposition pipeline.

As structure determination methods advance and the diversity of data used by integrative methods increases, PDB-IHM and RCSB.org will also continue to expand in scope, including improvements in the PDB data standards, deposition and curation workflows, validation methods, and visualization and analysis tools. These improvements will be informed and encouraged by collaborations across the structural biology community, in turn maintaining the PDB in service of structural biology as it maximizes its impact on cell biology and beyond.

## Data Availability

The RCSB Protein Data Bank (PDB) is available at RCSB.org.
